# Mindreading From the Eyes Declines With Aging – Evidence From 1,603 Subjects

**DOI:** 10.3389/fnagi.2020.550416

**Published:** 2020-10-22

**Authors:** Jana Kynast, Eva Maria Quinque, Maryna Polyakova, Tobias Luck, Steffi G. Riedel-Heller, Simon Baron-Cohen, Andreas Hinz, A. Veronica Witte, Julia Sacher, Arno Villringer, Matthias L. Schroeter

**Affiliations:** ^1^Department of Neurology, Max Planck Institute for Human Cognitive and Brain Sciences, Leipzig, Germany; ^2^LIFE – Leipzig Research Center for Civilization Diseases, University of Leipzig, Leipzig, Germany; ^3^Faculty of Applied Social Sciences, University of Applied Sciences Erfurt, Erfurt, Germany; ^4^Institute for Social Medicine, Occupational Medicine and Public Health, University Hospital Leipzig, Leipzig, Germany; ^5^Department of Psychiatry, Autism Research Centre, University of Cambridge, Cambridge, United Kingdom; ^6^Department for Medical Psychology and Sociology, University Hospital Leipzig, Leipzig, Germany; ^7^Clinic for Cognitive Neurology, University Hospital Leipzig, Leipzig, Germany

**Keywords:** aging, Reading the Mind in the Eyes Test, social cognition, women, men

## Abstract

Social cognition, in particular mindreading, enables the understanding of another individual’s feelings, intentions, desires, and mental states. The Reading the Mind in the Eyes Test (RMET) captures the ability to identify mental states from gaze. We investigated RMET accuracy in the context of age and cognition across the whole adult age-range (19–79 years) in a very large population-based sample (*N* = 1,603) with linear regression models accounting for cognitive abilities, neurological diseases, and psychiatric disorders. Higher age predicted lower RMET performance in women and men, suggesting difficulties to infer mental states from gaze at older age. Effects remained stable when taking other cognitive abilities and psychiatric disorders or neurological diseases into account. Our results show that RMET performance as a measure of social cognition declines with increasing age.

## Introduction

Theory of Mind (ToM) is an essential socio-cognitive ability that enables the attribution of mental states to self and others (Premack and Woodruff, 1978). The knowledge about another individual’s intentions, desires, and beliefs facilitates the prediction of future behavior, and enables a socially adequate reaction. Thus, ToM, also referred to as “mentalizing” (Morton and Johnson, 1991) or “mindreading” (Whiten, 1991), is essential for successful social interactions. On the contrary, deficits in ToM can compromise the initiation and maintenance of social relationships and individual well-being. ToM impairment can be found in several mental conditions, such as autism (Baron-Cohen et al., 1985, 1997, 2015; Brune and Brune-Cohrs, 2006), but also neurodegenerative diseases, like behavioral variant frontotemporal dementia (Gregory et al., 2002; Schroeter, 2012; Pardini et al., 2013; Schroeter et al., 2014, 2018), Alzheimer’s disease, and mild cognitive impairment (Baglio et al., 2012; Poletti and Bonuccelli, 2013). Recently, the Diagnostic and Statistical Manual of Mental Diseases, 5th edition (DSM-5; American Psychiatric Association, 2013) introduced social cognition as one core domain for the evaluation of cognitive performance in the context of dementia and its pre-stages, i.e., mild and major neurocognitive disorder, highlighting the clinical relevance of the reliable and effective assessment of ToM in adults.

The “Reading the Mind in the Eyes” Test (RMET; Baron-Cohen et al., 1997, 2001) is an established instrument for the neuropsychological assessment of socio-cognitive functions. It captures the ability to attribute mental states from gaze. The revised version includes 36 photographs of the eye-region of a person (Baron-Cohen et al., 2001). Out of four response options, the term most appropriately describing the pictured mental state shall be selected. Translated into many languages, the RMET is one of the most frequently used tasks to investigate ToM in adults (Kirkland et al., 2013). As the typical accuracy rate is usually below 100% (Pardini and Nichelli, 2009), the RMET is not limited by ceiling effects. The RMET has been discussed to tap mainly “hot” or “affective” ToM, which requires an understanding of others’ emotions, affective states, or feelings, whereas “cold” or “cognitive” ToM requires an understanding of their cognitive states, beliefs, thoughts, or intentions (Henry et al., 2013).

Recently, the influence of age on RMET accuracy has been in the focus of interest. Studies investigating age-related differences using cross-sectional data have shown a reduced RMET accuracy at older age (Pardini and Nichelli, 2009; Baglio et al., 2012; El Haj et al., 2016; Warrier et al., 2018). Other tests of ToM and social cognition show similar age effects. Henry et al. (2013) conducted a meta-analysis across six different types of ToM tasks, i.e., stories, eyes, videos, false belief-video, false belief-other, and faux pas, categorized into “affective” and “cognitive” ToM. Overall, older adults performed more poorly than younger adults with moderate magnitude. Remarkably, no effects for specific task parameters were detected.

Our study aimed at investigating the impact of age and several cognitive functions on the ability to identify mental states from gaze in the RMET in a large, population-based sample comprising 1,603 subjects aged 19–79 years. Based on literature findings (see above), we predicted better RMET performance in younger than older adults. Regarding cognitive abilities, RMET accuracy was hypothesized to be positively related to verbal intelligence (measured with a vocabulary test, and estimating individual vocabulary abilities) as suggested recently (Ahmed and Miller, 2011; Peterson and Miller, 2012; Baker et al., 2014). We further tested the impact of attention, memory, executive functions, and verbal fluency on RMET performance across the study sample, because age was reported to interact with these cognitive domains and RMET performance (Bailey and Henry, 2008; Duval et al., 2011; Dal Monte et al., 2014). Yet, the effect of domain-specific cognitive abilities on RMET accuracy has never been investigated in a large, community-dwelling sample covering the complete adult age range.

## Materials and Methods

### Psychological Tests

**FIGURE 1:**
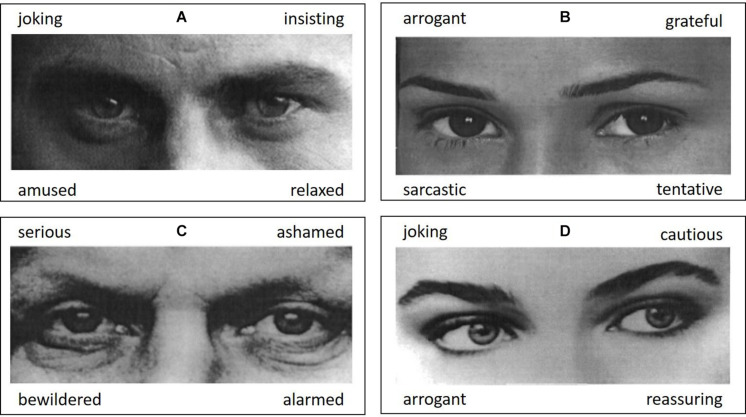
Example items from the Reading the Mind in the Eyes Test (RMET; Baron-Cohen et al., 2001; Bölte, 2005). For each photograph, the most appropriate mental state term shall be selected out of four response options. Correct responses are **(A)** insisting, **(B)** tentative, **(C)** serious, and **(D)** cautious. Pictures are taken from Bölte (2005).

This study was part of the adult study of the Leipzig Research Centre for Civilization Diseases (LIFE). The detailed study procedure is described elsewhere (Loeffler et al., 2015). ToM was assessed with the German version of the revised RMET (Baron-Cohen et al., 2001; Bölte, 2005). The test contains 36 pictures showing the eyes of either a man or a woman. Every picture is presented with four adjectives of which the one describing the pictured mental state best should be selected. Figure 1 illustrates four test stimuli. Computerized assessment was self-paced and needed approximately 10–15 min for completion. Additionally, all participants completed a 42-item test of verbal intelligence (vocabulary test, so called Wortschatztest, WST; Schmidt and Metzler, 1992). For each item, six words were presented. Of those, five were pseudo words and only one a real term existing in German. The latter shall be selected. For both tests (RMET, WST), accuracy rates (percentage of correct responses) were analyzed. Reaction times were not analyzed, as subjects were not explicitly instructed to respond as fast as possible. Furthermore, we avoided the well-known bias of speed slowing due to aging (Schroeter et al., 2003) by abstaining from analyses of reaction times.

### Study Cohort

All study participants were randomly selected from the population according to the study protocol (Loeffler et al., 2015). Of those randomly sampled citizens, where data had been provided by the resident’s registration office of the city of Leipzig, 10,000 participants were investigated in total. A subsample was characterized by in-depth cognitive phenotyping including, beside several other tests, the RMET. As the LIFE study was localized in the city of Leipzig (Saxony/Germany), most participants represented Caucasians from diverse social backgrounds. Information on age, and psychological measures (description below) was assessed.

Information on affiliation to either women or men was obtained directly from the resident’s registration office of the city of Leipzig, and, additionally, with a questionnaire given to the participants containing the question: “Which gender are you belonging to?” (In German “Welches Geschlecht haben Sie?”). The final study sample included 1,603 persons aged between 19 and 79 years (905 men; Table 1). Of note, both ways of determining affiliation to women or men showed a high coincidence, i.e., 100% of the study population with available information from both sources (93.4%) did show agreement on both factors. For the total LIFE population of 10,000 participants, this was the case for 99.93% (90% with both information). None of the six participants with disagreement between both measures in the whole LIFE study were included in our study. In the following, this factor – affiliation to women or men – will be characterized by the term gender. Please, find a critical discussion of this approach in the discussion section.

**TABLE 1:** Demographic characteristics and descriptive results of the Reading the Mind in the Eyes Test (RMET) and the vocabulary test (Wortschatztest, WST) for the whole sample (*N* = 1,603).

		**Gender**		**Independent sample *t*-test t(df;n), *p*-value**
		**Men**	**Women**	
*n*		905	698	
Age	*M* (SD)	60.26 (14.99)	60.35 (14)	*t*(1;1544) = −0.118, *p* > 0.05
WST (% correct)	*M* (SD)	78.48 (10.20)	74.92 (10.48)	*t*(1;1601) = 6.805, *p* < 0.001
RMET (% correct)	*M* (SD)	62.76 (10.54)	63.79 (10.51)	*t*(1;1601) = −1.956, *p* = 0.051

*Note: df, degrees of freedom; *M*, mean; *n*, number; and SD standard deviation. Number of subjects and gender distribution per age group are 25–29 years 34 men/17 women, 30–34 39/17, 35–39 33/23, 40–44 57/52, 45–49 61/52, 50–54 35/27, 55–59 33/32, 60–64 105/122, 65–69 191/138, 70–74 211/149, and 75–80 90/54.*

All individuals scored above chance level in either psychological test (WST > 17%; RMET > 25%), ensuring a sufficient understanding of the test instructions. Chance levels for each test have been calculated by relating the number of correct responses to the total number of responses provided for each item. Since in the RMET each item is presented with four response alternatives of which only one answer is correct, chance level is calculated as 1/4 (25%, respectively). In the WST, for each item six response alternatives are given of which only one is correct. The chance level is hence calculated as 1/6 (17%, respectively).

The study was approved by the ethics committee of the University of Leipzig and was in accordance with the latest version of the Declaration of Helsinki. Each subject provided written informed consent.

### Statistical Analysis

#### Linear Regression of Age and Gender on RMET Performance

The influence of age, gender and verbal intelligence on RMET performance was tested with a linear regression model predicting RMET accuracy (criterion) from age, gender, the interaction term age^∗^gender, and WST performance (predictors). We predicted older age to be linearly associated with lower RMET performance. The interaction term (age^∗^gender) tests whether age affects RMET performance differently in men and women. Considering findings from the literature, a small, positive relation between WST performance and RMET accuracy was expected. For all analyses, data fulfilled the requirements for parametric statistical testing. Statistical analyses were performed using IBM SPSS Statistics for Windows, Version 22.0 (IBM Corp, 2013). All graphs were created using Microsoft Excel (Microsoft, 2010) and Adobe Illustrator CS5 (Adobe Systems, 2010).

#### Analyses of Potentially Modulating Factors: Cognitive Performance

Especially when investigating age effects on mindreading ability, performance on other cognitive domains must be considered to test the possible influence of cognitive decline on mindreading performance at old age. Therefore, scores representative of individual performance on several cognitive domains (i.e., attention, executive function, memory, and verbal fluency; Lakens, 2013) were additionally included as predictors of RMET performance in the linear regression model. To compute cognitive domain scores, neuropsychological test scores were standardized to the mean of age- and gender-specific groups from the actual sample. Measures pertaining to a specific cognitive domain were further averaged to compound scores reflecting domain-specific cognitive performance (for a full description of included tests and procedures see Kynast et al., 2018). Analysis included all individuals of the current sample with full recordings of cognitive measures (*N* = 1,157; 698 men). For all analyses, data fulfilled the requirements for parametric statistical testing.

#### Analyses of Potentially Modulating Factors: Neuropsychiatric Diseases

The inclusion criteria for the analysis sample were broadly defined, as we aimed at investigating effects in a generic population. However, neurological diseases and psychiatric disorders potentially influence RMET accuracy besides age, gender and cognitive abilities. Accordingly, we re-tested effects in a selected subsample of the study cohort without such diseases/disorders. Individuals fulfilling one or more of the following criteria were excluded from these analyses: (a) history of neurological or psychiatric disorder, alcohol and substance abuse, dementia, (b) stroke, tumor or brain injury according to structural magnetic resonance imaging, (c) intake of psychoactive medication, (d) depression score > 20 in the Center of Epidemiologic Studies Rating Scale Depression (Radloff, 1977), and (e) extensive white matter hyperintensities exceeding Fazekas stage 1 as indices for small vessel disease (Fazekas et al., 1987; Kynast et al., 2018). Furthermore, subjects with severe cognitive dysfunction, i.e., dementia (operationalized as domain specific test performance below -2 standard deviation of the age group mean, DSM-5; American Psychiatric Association, 2013) were excluded. All analyses have been re-calculated within this selected subsample (*N* = 978; 576 men; age *M* = 58.13 years), including (1) linear regression analysis predicting RMET performance from age, gender, age^∗^gender and WST performance, (2) linear regression analysis predicting RMET performance from age, gender, age^∗^gender, attention, executive function, memory, verbal fluency, and WST performance.

## Results

### Test Performance

**FIGURE 2:**
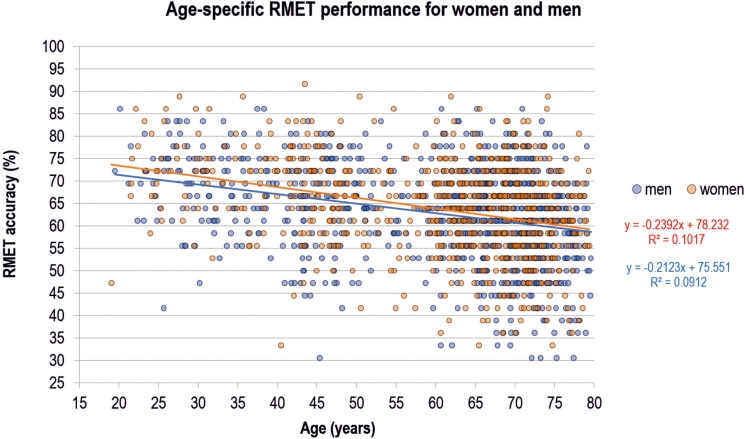
Reading the Mind in the Eyes Test (RMET) performance of women and men across age. Each dot represents one individual from the analysis sample (whole sample, *N* = 1,603). Linear regression lines for the prediction of RMET performance from age and equations are shown additionally.

Figure 2 shows individual RMET performance scores for women and men in relation to age for the whole sample. Performance declined with aging in both women and men on a descriptive level. Mean age, WST performance reflecting verbal intelligence, and RMET performance in men compared to performance in women are listed in Table 1. Men scored significantly higher in the WST compared to women (*p* < 0.001), whereas the effect of gender on RMET performance just missed the significance threshold (*p* = 0.051).

### Linear Regression Analyses

The linear regression model including age, gender, age^∗^gender, and WST performance as predictors of RMET performance was significant [*F*(4;1598) = 78.142, *p* < 0.001] [results are reported as *F* (degrees of freedom; number of subjects)]. Older age (*b* = −0.208, *p* < 0.001) and lower verbal intelligence (*b* = 0.261, *p* < 0.001) were significantly related to lower RMET accuracy. No significant results were obtained for gender (*b* = 2.677, *p* = 0.201) and the interaction term gender^∗^age (*b* = −0.011, *p* = 0.734). The statistical model explained 16.4% of variance in RMET accuracy (*R*^2^ = 0.164).

### Analyses of Potentially Modulating Factors: Cognitive Performance

Results for this analysis are illustrated in Table 2. The linear regression model including age, gender, age^∗^gender, attention, executive functions, memory, verbal fluency, and WST performance as predictors of RMET performance was significant [*F*(8;1148) = 30.088, *p* < 0.001, and *R*^2^ = 0.145]. Besides age (*p* < 0.001) and WST performance (*p* < 0.001), memory (*p* = 0.037) was a significant predictor of RMET performance (Table 2).

**TABLE 2:** Linear regression analysis to predict Reading the Mind in the Eyes Test (RMET) performance from age, gender, and domain-specific cognitive scores (whole sample, *N* = 1,157).

	***B***	***SE***	***b***	***p***
Age	–0.214	0.024	–0.297	<0.001
Gender	2.957	2.274	0.137	0.232
Age*Gender	–0.005	0.04	–0.015	0.897
WST	0.217	0.03	0.214	<0.001
Attention	0.710	0.377	0.053	0.06
Executive function	–0.093	0.412	–0.006	0.822
Memory	0.914	0.438	0.062	0.037
Verbal fluency	0.306	0.358	0.026	0.393

*Note: *B*, regression coefficient (non-standardized); *SE*, standard error; *b*, regression coefficient (standardized); and WST, Wortschatztest (vocabulary test). The statistical model explains 14.5% of the variance in RMET performance corresponding to *R*^2^ of 0.145 (*p* < 0.001).*

### Analyses of Potentially Modulating Factors: Neuropsychiatric Diseases

**FIGURE 3:**
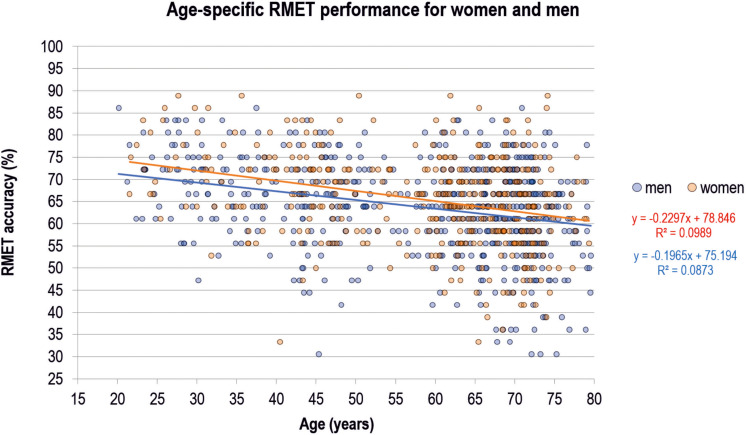
Reading the Mind in the Eyes Test (RMET) performance of women and men across age. Each dot represents one individual from the selected subsample after exclusion of neurological diseases or psychiatric disorders (*n* = 978). Linear regression lines for the prediction of RMET performance from age and equations are shown additionally.

Linear regression analyses performed on the selected subsample without neurological diseases or psychiatric disorders (*N* = 978) revealed generally comparable results to analyses performed on the entire sample. Respective results are presented in Figure 3 and Tables 3, 4. Accordingly, results of the first analysis were confirmed and were not related to possibly confounding factors such as neurological diseases or psychiatric disorders. Whereas the association between RMET performance and age as well as verbal intelligence was replicated in the linear regression analysis, the association with memory disappeared.

**TABLE 3:** Linear regression analysis to predict Reading the Mind in the Eyes Test (RMET) performance from age, gender, and WST performance in the selected subsample after exclusion of neurological diseases or psychiatric disorders (*n* = 978).

	***B***	***SE***	***b***	***p***
Age	–0.193	0.026	–0.279	<0.001
Gender	3.509	2.572	0.167	0.173
Age*Gender	–0.018	0.043	–0.053	0.671
WST	0.224	0.032	0.215	<0.001

*Note. *B*, regression coefficient (non-standardized); *SE*, standard error; *b*, regression coefficient (standardized); and WST, Wortschatztest (vocabulary test). The statistical model explains 13.8% of the variance in RMET performance corresponding to *R*^2^ of 0.138 (*p* < 0.001).*

**TABLE 4:** Linear regression analysis to predict Reading the Mind in the Eyes Test (RMET) performance from age, gender, and domain-specific cognitive scores in the selected subsample after exclusion of neurological diseases or psychiatric disorders (*n* = 978).

	***B***	***SE***	***b***	***p***
Age	–0.191	0.026	–0.28	<0.001
Gender	3.693	2.646	0.714	0.163
Age*Gender	–0.018	0.044	–0.415	0.678
WST	0.204	0.035	0.192	<0.001
Attention	0.414	0.436	0.031	0.342
Executive function	0	0.464	0	1
Memory	0.456	0.476	0.032	0.338
Verbal fluency	0.412	0.394	0.035	0.296

*Note: *B*, regression coefficient (non-standardized); *SE*, standard error; *b*, regression coefficient (standardized); and WST, Wortschatztest (vocabulary test). The statistical model explains 13.8% of the variance in RMET performance corresponding to *R*^2^ of 0.138 (*p* < 0.001).*

## Discussion

The RMET is one of the most frequently used tools for the assessment of ToM in adults. The primary aim of our study was testing the effects of age on RMET accuracy in a lifespan perspective. Therefore, we investigated mindreading from the eyes in a population-based lifespan sample including more than 1,600 randomly selected individuals. Crucially, we considered the potential impact of distinct cognitive functions on the association between the ability to identify mental states from gaze, and age. We found a linear relation between RMET accuracy and age. Older age was associated with lower RMET accuracy in both men and women. This is in line with other studies that have investigated this issue. In sum, our results indicate an age-related decline in RMET performance.

It has been discussed previously if changes of ToM at older age may be specifically related to stimuli and modalities (Henry et al., 2013). Thus, the age-related performance drop in mindreading from the eyes may be related to test-specific characteristics. The RMET is an advanced test of ToM that focuses on the eyes as social cues. Thus, a decrease in test accuracy at older age may potentially be driven by a specific impairment in extracting mental state information when social cues are limited in general, and/or this limitation pertains specifically to the eyes. Older adults may prefer other social cues, such as the mouth, body posture, or voice (Phillips et al., 2014), or rely on the integration of information from multiple social sources (Hunter et al., 2010).

Additional aspects that must be considered in this context include stimulus characteristics. Individual features of the interaction partner, such as age, gender or ethnicity, have been shown to influence social information perception and processing, and behavior (Seidel et al., 2010; Slessor et al., 2010). Consequently, the features of the RMET stimuli (i.e., gender, age, and emotional valence) might potentially impact test performance differently in young and older adults (Kynast and Schroeter, 2018). Thus, elderly individuals may show deficits in accurately determining a young person’s mental state compared to an older person’s mental state (i.e., an in-group bias). Also, emotional complexity may be an important factor, as shown in a recent study (Duval et al., 2011). There, older participants performed as accurate as the young group when confronted with more basic emotions in the RMET but had specific difficulties with complex emotions. The “positivity effect,” i.e., that older adults show a relative preference for positive over negative material in cognitive tasks (Kennedy et al., 2004; Phillips et al., 2014), may also apply to the RMET and explain lower accuracy rates compared to younger adults. However, these effects need further investigation and may be subject to future studies.

Considering cognitive abilities associated with RMET performance, a significant relation was found with both vocabulary test performance (WST) and memory. This may be best explained by the fact that these measures strongly overlap in their focus on verbal content and, thus, strongly address verbal abilities. A conceptual representation of the response alternatives given with each RMET item is required for target word selection (Baron-Cohen et al., 1997), i.e., the individual must know what the response alternatives mean to select the most appropriate term. Consequently, a comprehensive vocabulary potentially facilitates proper task completion. The results verify this assumption by indicating a relative importance of verbal abilities for mindreading from the eyes.

Although this study was carefully conducted, several limitations must be discussed. In accordance with previous studies in the field, we applied a rather conventional binary concept of sex/gender, i.e., affiliation to women or men, to characterize men’s and women’s ability to identify mental states from gaze. This approach, which refers rather to gender than to biological sex, enables comparability with previous studies but is a general limitation since further biological and environmental characteristics impacting individual self-concepts of sexual identity (e.g., social roles, experiences, etc.; for review see Jäncke, 2018) were not investigated and, thus, potential effects thereof on mindreading remain still unknown. Moreover, Hyde et al. (2019) described five challenges that generally question the so called gender binary, i.e., that humans comprise only two types of beings, women and men: Neuroscience and neuroendocrinology questioning sexual brain/hormonal dimorphism (see also Jäncke et al., 2015), psychology underlining rather similarities than differences between women and men on a broad continuum (as also suggested by our data), research on transgender and non-binary individuals’ identities, and research suggesting gender/sex as binary category to be culturally determined. Accordingly, Hyde (2005) postulated the gender similarities hypothesis – based on meta-analytical evidence – that males and females are similar on most psychological variables. To expand the view within social cognitive research, future studies must incorporate a broader definition beyond the conventional concept of sex/gender to fully picture the diversity in sexual identity and its impact on cognitive processes (see Ainsworth, 2015; Dotson and Duarte, 2020). Due to the conceptual limitations of our study regarding this parameter we abstained here from discussing our results in depth.

Moreover, one may criticize that our results are potentially confounded by neurological diseases or psychiatric disorders. We can exclude such a bias, because we confirmed our results in a selected subsample, where we excluded relevant subjects. Note, that confining the cohort with this strategy also reduced the relatively high variability in the uncontrolled cohort as shown in Figures 2, 3. Furthermore, the number of participants was not balanced across the different age groups, which can be regarded statistically less-than-ideal. This difference is related to the conceptual development of the LIFE study with a main focus on the elderly age ranges. However, numbers in younger age groups are still sufficient and large if compared to earlier studies on this issue, justifying regarding results as reliable. Requirements for the application of parametric statistical methods were tested carefully. Results of our cross-sectional study shall be proven in future longitudinal studies also involving other languages and ethnicities beside our German version applied in a Caucasian cohort, and including a comprehensive conceptualizing of sex/gender.

In summary, our study investigated the effects of age, and domain-specific cognitive abilities on mental state recognition from gaze in the RMET in a population-based sample including 1,603 adults aged 19–79 years, i.e., across the whole lifespan. Results reveal declining performance in the RMET with aging for both, women and men. Taken together, ToM, and particularly mindreading from the eyes might be interpreted as a universal human feature present in both men and women over the whole lifespan but declining with aging.

## Data Availability Statement

Data are available from the authors upon reasonable request.

## Ethics Statement

The studies involving human participants were reviewed and approved by Ethics Committee of the University of Leipzig. The participants provided their written informed consent to participate in this study.

## Author Contributions

JK planned and conducted statistical analyses, wrote the first draft of the manuscript including creation of the figures, and modified all subsequent drafts. EQ planned the study, assessed and processed data, and substantially contributed to all drafts of the manuscript. MP was responsible for data quality management, and contributed substantially to all drafts of the manuscript. TL, SR-H, and AV designed the study, interpreted results, and reviewed the final draft of the manuscript. SB-C, AH, JS, and AW contributed substantially to the interpretation of the results. MS designed the study, supervised data acquisition and analyses, substantially contributed to the interpretation of the results and made substantial modifications to all drafts of the manuscript. Further, he took the lead in revising the manuscript. All authors approved the final version of the manuscript.

## Conflict of Interest

The authors declare that the research was conducted in the absence of any commercial or financial relationships that could be construed as a potential conflict of interest.

## Abbreviations

LIFE: Leipzig research Centre for Civilization Diseases
RMET: Reading the Mind in the Eyes test
ToM: Theory of Mind
WST: Wortschatztest.
